# Crotonylation-related gene *GCDH* promotes osteoarthritis pathogenesis through flavin adenine dinucleotide signaling: mechanism exploration and experimental validation

**DOI:** 10.3389/fnut.2025.1646005

**Published:** 2026-01-06

**Authors:** Jingkai Di, Zijian Guo, Yijing Di, Luyi Zhang, Yingda Qin, Feida Wang, Chuan Xiang

**Affiliations:** 1 Department of Orthopedics, Second Hospital of Shanxi Medical University, Taiyuan, China; 2 Shanxi Key Laboratory of Bone and Soft Tissue Injury Repair, Taiyuan, China; 3 The First Hospital of Shanxi Medical University, Taiyuan, China; 4 The Second Hospital of Shanxi Medical University, Taiyuan, China

**Keywords:** osteoarthritis, post-translational modification, crotonylation, chondrocyte, mediation

## Abstract

**Introduction:**

Osteoarthritis (OA) is a prevalent degenerative joint disorder characterized by progressive cartilage degradation and synovial inflammation. This study is designed to elucidate the role of crotonylation genes in OA progression.

**Materials and methods:**

The crotonylation genes exposure and OA outcome data were obtained from the eQTLGen consortium and UK Biobank databases, respectively. Mendelian randomization analysis was employed to establish mechanistic links between crotonylation genes and OA, with subsequent validation conducted through cartilage RNA sequencing (RNA-seq) data. GCDH expression and its regulatory effects on key extracellular matrix (ECM) markers were assessed by Western blot (WB) and quantitative real-time polymerase chain reaction (qRT-PCR) in OA chondrocytes. Moreover, CCK-8, EdU and Transwell assays were utilized to assess differences in chondrocyte proliferation and migration potential at the time of OA versus after *GCDH* knockdown. Finally, downstream mechanisms were explored using mediation analysis.

**Results:**

The study identified *GCDH* as a risk factor contributing to OA susceptibility (OR = 1.048, 95%CI = 1.004–1.093). RNA-seq, qRT-PCR and WB consistently demonstrated significant upregulation of *GCDH* expression in OA. Furthermore, *GCDH* was identified as a critical regulator of ECM metabolism in OA pathogenesis. In addition, functional experiments showed that the proliferation and migration ability of chondrocytes was restored in the OA group after *GCDH* knockdown. Finally, the exploration of the downstream mechanism showed that the flavin adenine dinucleotide mediated the above process.

**Conclusion:**

The crotonylation gene *GCDH* was identified as a potential risk factor for OA pathogenesis, thus providing a novel molecular target and interventions for the development of targeted OA therapeutic strategies.

## 1 Introduction

Osteoarthritis (OA) represents a prevalent degenerative joint disorder involving progressive deterioration of articular cartilage, accompanied by synovial membrane inflammation and pathological alterations in subchondral bone architecture, which primarily manifests as joint pain, stiffness and restricted movement (1–3). With the aging population and rising obesity rates, the global disease burden of OA is increasing. It has been reported that the number of people with OA globally is expected to exceed 952 million by 2050, bringing a heavy socioeconomic burden (4, 5). However, current OA treatments (e.g., oral drugs such as non-steroidal anti-inflammatory drugs) remain confined to alleviating symptoms (6). Therefore, in-depth analysis of OA pathological mechanisms and exploration of new therapeutic and early intervention targets has become critical breakthroughs to mitigate the escalating public health burden of OA in aging populations.

Post-translational modifications (PTMs) regulate the functional heterogeneity of proteins through dynamic site-specific amino acid modifications (7). Imbalances in PTM networks are closely associated with pathological processes, establishing them as potential molecular targets for disease treatment (8). Among various PTM types, lysine crotonylation (Kcr) has garnered significant attention due to its unique metabolic regulatory properties (9). This modification is primarily mediated by crotonyltransferases that transfer crotonyl from crotonyl-CoA to lysine residues on target proteins (10). It has been shown that this modification is widely involved in the development of several diseases, such as cancer (11). Specifically, aberrant enrichment of the Kcr modification site H3K18 in the transcriptional start region of the small intestinal epithelium has been reported to promote cancer cell proliferation, thereby driving colorectal cancer progression (12). Furthermore, in a randomized controlled trial involving patients with hypertrophic cardiomyopathy, it was demonstrated that the upregulation of two specific Kcr sites, H3K18cr and H2BK12cr, within cardiomyocytes significantly enhances the recruitment of the transcription factor NFATc3 to promoter regions. This interaction is recognized as a crucial factor contributing to cardiomyocyte hypertrophy (13). Until now, the regulatory mechanisms of Kcr in various diseases have been progressively elucidated (14, 15). Nevertheless, the contribution of crotonylation in the pathogenesis and progression of OA remains to be fully elucidated, thus exploring the potential relationship between Kcr and OA is of great research significance. As an emerging epigenetic modification, the role of protein crotonylation in OA pathogenesis remains largely unexplored. Although its direct mechanisms are not fully defined, growing evidence implicates it in OA pathology. Intra-articular injection of crotonyl-CoA has been shown to induce the release of inflammatory cytokines TNF-*α* and IL-1β and provoke significant pain behaviors (16). This suggests that crotonylation may upregulate pro-nociceptive factors, positioning it as a potential key mediator of OA-associated pain and functional disability. Furthermore, Han et al. demonstrated that elevated crotonylation levels promote osteogenic differentiation of mesenchymal stem cells (17). This discovery indicates that crotonylation drives the pathological calcification of articular cartilage through its ability to promote transdifferentiation toward an osteoblastic phenotype, thereby facilitating ectopic mineralization. Therefore, elucidating the functional roles and regulatory networks of crotonylation-related genes is crucial for a comprehensive understanding of OA pathogenesis.

In our research, data from UK Biobank (UKB) and eQTLGen Consortium were integrated to conduct causal inference analyses and identify potential associations between crotonylation-related genes and OA (18, 19). Additionally, the strongly associated genes were further validated using RNA sequencing (RNA-seq) data from human knee cartilage tissues, as well as through expression and functional experiments. Finally, the causal mechanisms of downstream signaling pathways were explored. This research sought to elucidate the connection between OA and crotonylation genes, thereby providing new perspectives for identifying new therapeutic targets and treatment strategies for OA.

## 2 Materials and methods

### 2.1 Research framework

**Figure 1:**
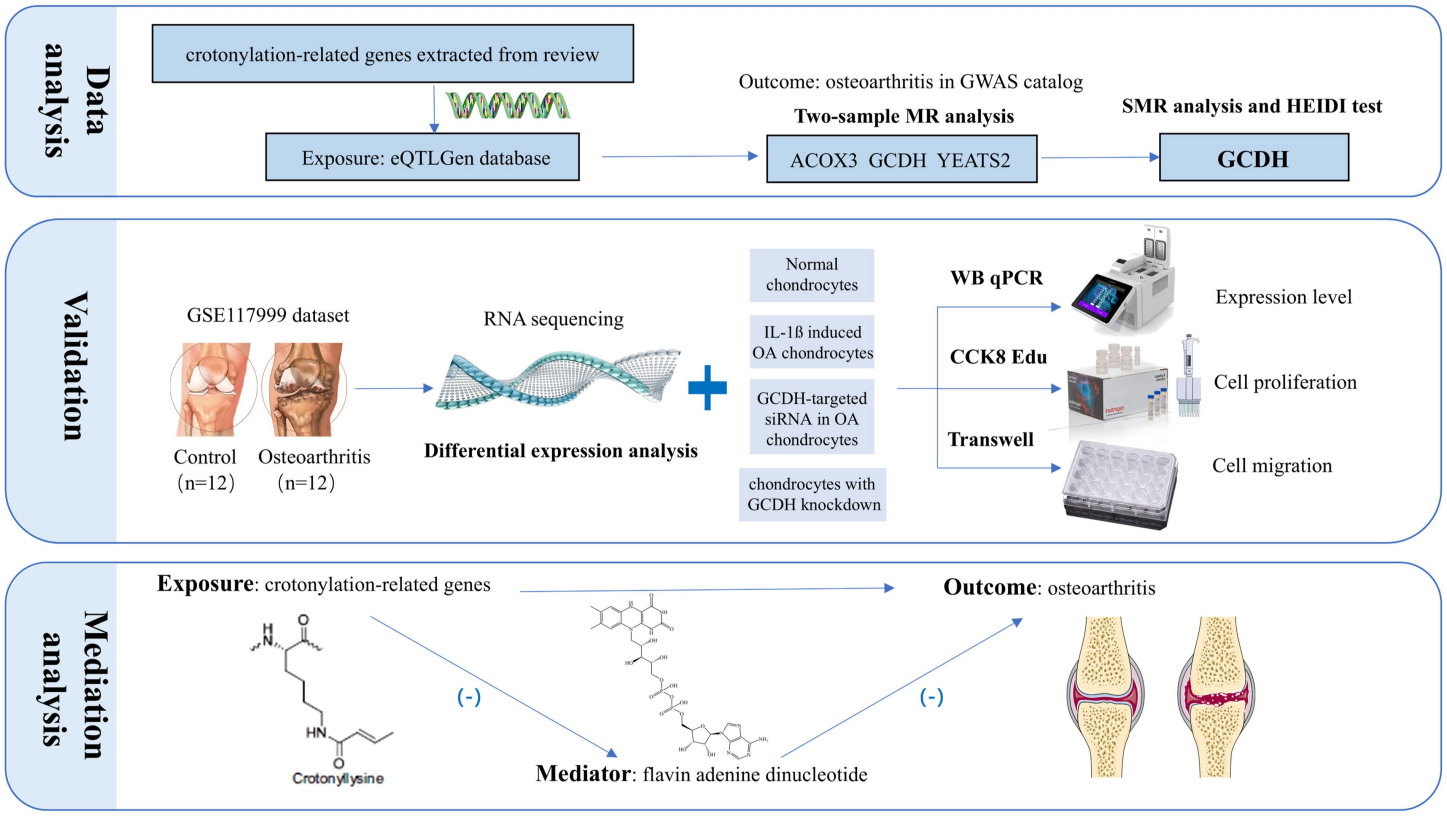
The research workflow proceeded as follows: First, a dual-validation approach integrating MR and SMR analyses pinpointed GCDH as a putative OA risk gene among crotonylation-related candidates. Following this, we validated GCDH expression at the transcriptional and protein levels via RNA-seq, qRT-PCR, and Western blot analysis, and further assessed its role in regulating chondrocyte extracellular matrix metabolism. Building on this, functional assessments through CCK-8, EdU, and Transwell assays elucidated the critical role of GCDH in chondrocyte proliferation and migration. The final step involved mechanistic exploration, which uncovered the plasma metabolite FAD as a key downstream mediator in the GCDH-driven OA pathway. MR, Mendelian randomization; SMR, summary-data-based Mendelian randomization; GCDH, Glutaryl-CoA Dehydrogenase; OA, osteoarthritis; qRT-PCR, quantitative real-time polymerase chain reaction; EdU, 5-Ethynyl-2′-deoxyuridine; CCK8, Cell Counting Kit-8.

This research seeks to explore the potential association between the upregulation of crotonylation genes and the onset and progression of OA, as well as to investigate the downstream mechanisms involved (Figure 1). The crotonylation genes were derived from the research by Yang et al. (20), which involved a total of 34 related genes. Subsequently, we screened 16 crotonylation-related genes by eQTLGen database (*ACADS, ACOX1, ACOX2, ACOX3, ACSS2, DPF2, GCDH, HDAC1, HDAC3, HDAC7, KAT2B, SIRT1, SIRT2, SIRT3, SIRT6, YEATS2*). First, the study combined the expression quantitative trait loci (eQTLs) of these genes with the genome-wide association studies (GWAS) data on OA for two-sample Mendelian randomization (MR) and summary-data-based MR (SMR) analyses, which led to the identification of crotonylation genes with strong causal association with OA. Additionally, RNA-seq data from human knee cartilage and functional and expressivity experiments were utilized to further validate the conclusions. Finally, we explored the specific downstream mechanisms by which crotonylation genes influence OA progression.

### 2.2 Data source

The exposure data for this study were derived from the eQTLGen Consortium and contained summary information on genetic variation in crotonylation genes. The data, based on 31,684 healthy European ancestry samples, covered cis-eQTL information in blood and peripheral blood mononuclear cells, involving a total of 16,987 genes. During the data collection process, relevant variations in the sex chromosome and mitochondrial DNA (mtDNA) were excluded (18). Meanwhile, OA related outcome data came from the UKB, a study of white Europeans aged 40–69 years, involving 31,267 clinically diagnosed OA cases and 184,763 healthy control samples (19). In addition, among the downstream mechanisms, we mainly explored the roles of metabolites, utilizing data from 1,091 blood metabolites and 309 metabolite ratios across 8,091 individuals (21). The above summary statistics are available from publicly available websites (22, 23).

### 2.3 Statistical analysis

#### 2.3.1 Selection of genetic instruments

We included all genetic instrumental variables (IVs) that met stringent quality control criteria to ensure the robustness of the instruments. First, GWAS-identified significant single nucleotide polymorphisms (SNPs) were considered as IVs strongly associated with OA (*p* < 5 × 10^−8^). Subsequently, to avoid bias caused by linkage disequilibrium (LD), we performed cluster analysis utilizing data from the 1,000 Genomes Project of European descent, considering a window of 10,000 kp and *r*^2^ > 0.001 as indicators of strong linkage on the genetic sequence. Additionally, SNPs failing to reach the minor allele frequency (MAF) threshold of 0.01 were filtered out to remove the interference of rare variants on the results (24). Finally, we selected strong instruments with an F-statistic exceeding 10.

#### 2.3.2 Two-sample Mendelian randomization

The STROBE-MR guidelines were used to enhance the integrity of our study (Supplementary material 1). Two-sample MR approach was utilized in our research to explore the potential of crotonylation genes as therapeutic or risk factors for OA. The inverse-variance weighted (IVW) was chosen as the primary approach for analysis, and Cochran’s Q test was utilized to evaluate the consistency between the IVW and MR-Egger methods. Additionally, the intercept of the MR-Egger regression represented potential directional pleiotropy among multiple IVs (25). To further detect and adjust for SNPs that might be outliers due to horizontal pleiotropy, we employed the MR-PRESSO approach. SNPs identified as non-pleiotropic were incorporated into the ultimate MR analysis. Finally, Leave-one-SNP-out examination was employed, where each SNP was eliminated to identify the potential impact of single SNPs on the overall causal effect. The analyses were performed using the “TwoSampleMR” and “MRPRESSO” software packages, with *p* < 0.05 considered statistically significant.

#### 2.3.3 Summary-data-based Mendelian randomization analysis

The SMR model applies the MR principle to jointly analyze the multiple associations between gene expression levels of specific protein targets and phenotypes caused by shared variations at gene loci (26). In the current research, it was utilized to further validate crotonylation genes strongly correlated with OA, with results considered significant at *p* < 0.05. It is noteworthy that significant SMR results may also reflect interlocking models with lower biological relevance. Therefore, the heterogeneity in dependent instruments (HEIDI) test was utilized to distinguish between multiple influences of genetic variations on phenotypes and associations caused by LD. Causal associations with a *p* < 0.05 in the HEIDI test were deemed potentially driven by pleiotropy, and therefore were subsequently excluded from further analyses. Both SMR and the HEIDI test were implemented with SMR software (version 1.03).

### 2.4 Differentially expressed genes analysis based on RNA-seq

We extracted RNA-seq data of human knee cartilage from the GSE117999 dataset in the Gene Expression Omnibus (GEO) database.^1^ This dataset comprises 12 clinical cases diagnosed with OA and 12 healthy control tissues, in which cartilage samples were taken from the smooth surface of the non-weight-bearing region of the medial intercondylar notch. Our research recognize differentially expressed genes (DEGs) in normal and OA-affected cartilage tissue samples employing the “Limma” R package (27). Furthermore, gene with a false discovery rate (FDR) above 0.05 was filtered out in order to reduce the likelihood of false-positive results from repeated testing. Stringent filtering was applied to define DEGs, requiring an FDR < 0.05 (Benjamini-Hochberg adjusted) and an absolute log2 (fold change) ≥ 0.585 to reduce the likelihood of false-positive discoveries.

### 2.5 *In vitro* cellular experiments

#### 2.5.1 Cell culture and IL-1β induction

In the present research, the immortalized human cartilage cell line IM-H488 was obtained from IMMOCELL (Xiamen, China). The cells were cultured in DMEM/F12 culture fluid supplemented with 10% fetal bovine serum (v/v) and 1% penicillin/streptomycin (v/v). Chondrocytes were digested and passaged every 48–72 h utilizing 0.125% trypsin–EDTA (diluted in PBS). Cells were induced by adding IL-1β to the wells for 16 h to construct OA group chondrocytes. To maintain phenotypic stability of the study, we used cells prior to passage 2.

#### 2.5.2 Cell transfection

A total of 2 × 10^6^ chondrocytes, during the period of exponential growth, were seeded onto a 6-well culture plate. Upon achieving approximately 70% confluence, Lipofectamine™ 2000 transfection reagent was utilized to introduce specific siRNA targeting Glutaryl-CoA dehydrogenase (*GCDH*) gene, along with the corresponding control from the Public Protein/Plasmid Library located in Nanjing, Jiangsu Province, China. Following the transfection, the cells were incubated for 8 h to ensure optimal binding between the siRNA and the target mRNA, thereby facilitating effective gene knockdown for subsequent experimental analyses. The effectiveness of the knockdown of the GCDH gene was subsequently assessed using Quantitative real-time polymerase chain reaction (qRT-PCR) and Western Blot (WB).

#### 2.5.3 CCK8 assay

The CCK-8 assay was employed to evaluate chondrocyte multiplication (28). Chondrocytes were introduced into 96-well plate at 1 × 10^4^ cells each well (200 μL/well), followed by incubation at 37 °C for 24 h to promote cell adhesion. Following treatment, each well received 10 μL of CCK-8 solution combined with 90 μL of DMEM, and this was incubated for 1 h. Absorbance measurements were taken at 450 nm utilizing the SpectraMax M5 enzyme reader (Molecular Devices, United States).

#### 2.5.4 Edu assay

The chondrocyte multiplication was further assessed with the BeyoClick™ EdU Cell Proliferation Kit with Alexa Fluor 488 (Beyotime, China). Approximately 1 × 10^5^ chondrocytes were introduced into a 12-well plate. Each well was treated with EdU reagent (Beyotime, C0071S) for 2 h. Following incubation, the cells were rinsed three times in PBS and subsequently fixed and permeabilized with 4% paraformaldehyde solution (Dingguo Biotechnology, AR-0211) and 0.3% Triton X-100 (GenStar, VA11410). Afterward, the cells were subjected to incubation with the click reaction reagent in darkness at a temperature of 25 °C for a duration of 30 min. Finally, Hoechst 33342 reagent was added to label the nuclei. The samples were observed utilizing a Nikon ECLIPSE Ti-S fluorescence microscope, and results were obtained using NIS-Elements F v4.0 software.

#### 2.5.5 Transwell assay

A total of 1 × 10^6^ chondrocytes were introduced into 100 μL of serum-free culture solution and subsequently positioned in the top compartment of a 24-well plate. Meanwhile, 600 μL of complete medium was introduced into the bottom compartment to supply the essential growth factors for chondrocyte migration. After a 12-h maturation at 37 °C, the chondrocytes that traversed to the lower compartment were fixed using 4% paraformaldehyde for 15 min, followed by staining with a 1% crystal violet solution. Following a designated binding period, the excess staining solution was washed away utilizing phosphate-buffered saline (PBS). The stained migrating cells in the lower chamber were then imaged using fluorescence microscopy (Leica, Germany), and the number of chondrocytes was quantified.

#### 2.5.6 qRT-PCR

Total RNA was extracted from chondrocytes using TRIzol reagents (Invitrogen, Carlsbad, CA). The purified RNA was then converted into complementary DNA (cDNA) using the PrimeScript RT kit (Takara, Chiga, Japan). Following this, qRT-PCR was performed using the SYBR Green RT-PCR kit (Takara, Chiga, Japan) and the QuantStudio™ 6 Flex real-time fluorescence quantitative PCR system (Thermo Fisher, United States). The amplification process was monitored in real-time by detecting changes in fluorescence intensity. The sequences of the primers used are available in the supplementary file (Supplementary material 2).

#### 2.5.7 Western blot

Protein was isolated from chondrocytes using a lysis buffer supplemented with protease inhibitors (AR0102, Boster, Wuhan, China). The BCA assay kit (AR1189, Boster, Wuhan, China) was employed for the quantitative assessment of protein concentration. Following this, the proteins were separated through constant pressure and constant current electrophoresis and then moved to a PVDF membrane (Millipore, United States). The membrane was then blocked with 5% skimmed milk. ADAMTS5 Rabbit pAb (A2836; 1:1,000), Aggrecan Rabbit pAb (A8536;1:1,000), COL2A1 Rabbit pAb (A1560; 1:1,000), MMP13 Rabbit pAb (A11755; 1:1,000), GCDH Rabbit pAb (A9057; 1:1,000) and SOX9 Rabbit pAb (A2479; 1:1,000) were added to the membrane, and all were provided by Abclonal (Wuhan, China) and incubated at 4 °C overnight. Following three washes of the membrane with TBST, Goat Anti-Rabbit IgG (H + L) (BA1055, Boster, Wuhan, China) was introduced and permitted to incubate at 25 °C for 2 h, along with *β*-actin rabbit mAb (AC026; 1:100,000) as an internal control, also provided by Abclonal (Wuhan, China). The protein bands were detected with FG Super Sensitive ECL Luminescence Reagent (MA0186, Meilunbio, Dalian, China). Protein expression levels were detected with a ChemiDoc XRS + Gel Imaging System (BIO-RAD, California, United States). The relative expression levels of the proteins were calculated utilizing the ratio of the intensity of the target bands to those of the endogenous reference bands.

### 2.6 Downstream mechanism

To elucidate how the crotonoylation-related genes influence OA through specific metabolic mechanisms, our study applied a two-step MR approach. Specifically, the first step evaluated the causal effect between crotonoylation-related genes and plasma metabolites, resulting in a β1 value. The second step estimates the effect of the identified intermediate plasma metabolites on OA, resulting in β2. β3 represents the total effect of crotonylation genes on OA. The analysis evaluated the associated effects of each step separately and explored how crotonylation genes influence the onset of OA through mediating variables. The formula “β1_2 = β1 * β2” was utilized to evaluate the indirect effect of mediating factors on OA, and “β1 * β2/ β3” was utilized to calculate its mediating ratio.

## 3 Results

### 3.1 Identification of candidate genes for OA

**Figure 2:**
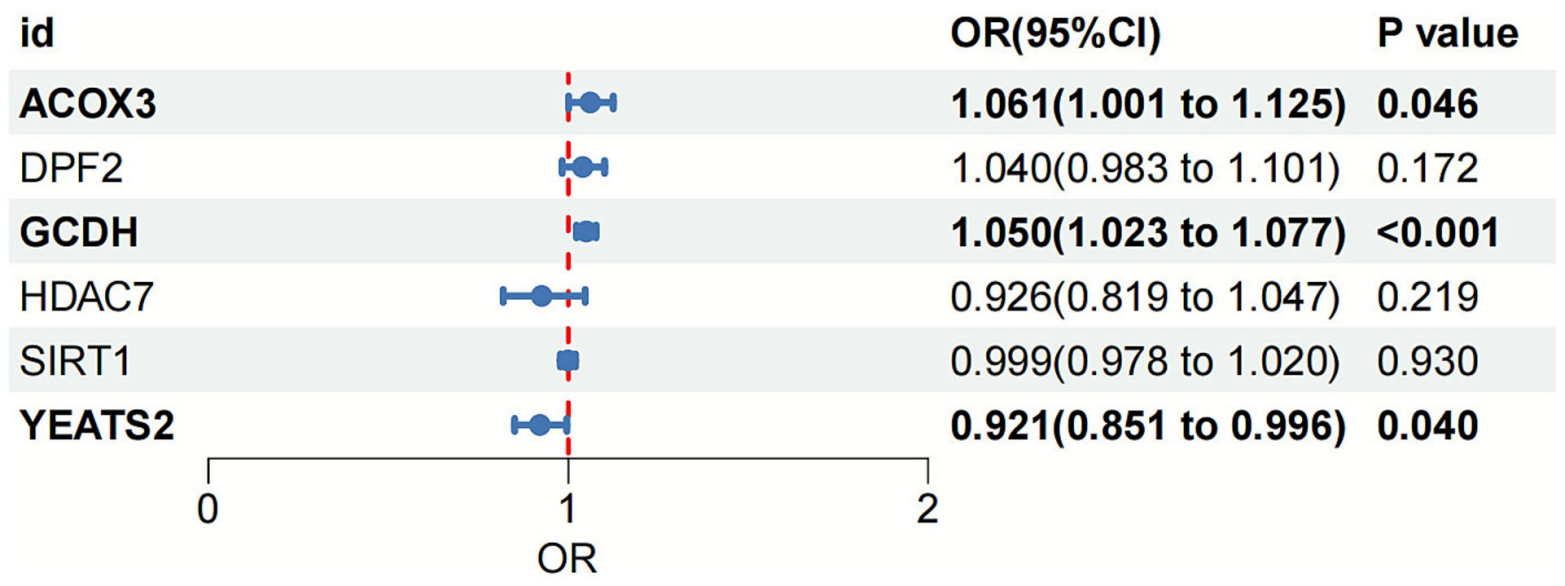
Forest plot of the association between crotonylation genes and OA based on MR analysis. OA, osteoarthritis; MR, Mendelian randomization.

Two-sample MR analysis suggested that *ACOX3*, *GCDH*, and *YEATS2* might be causally associated with OA as risk factors for its pathogenesis (*ACOX3*: OR = 1.061, 95%CI = 1.001–1.125; *GCDH*: OR = 1.050, 95%CI = 1.023–1.077; *YEATS2*: OR = 0.921, 95%CI = 0.851–0.996) (Figure 2). The reliability of the findings was verified by tests of heterogeneity and horizontal pleiotropy (*p* > 0.05). Additionally, the leave-one-out method revealed no individual SNP demonstrated dominant effects on the outcomes (Supplementary material 3).

### 3.2 SMR and HEIDI test between GCDH in gene expression and OA

Further, SMR analysis was employed to perform dual validation of potential associations between the genetic variants of strongly correlated genes identified in the previous step and OA. The results demonstrated that upregulated expression of *GCDH* remained associated with a promoting effect on OA risk (OR = 1.048, 95% CI = 1.004–1.093). Additionally, The HEIDI analysis demonstrated this relationship was not driven by LD (p-HEIDI > 0.05) (Table 1). No significant association with OA was observed for either *ACOX3* (*p* = 0.820) or *YEATS2* (*p* = 0.383). This result drove the decision to focus subsequent investigations on GCDH.

**Table 1:** The SMR and HEIDI tests show the association of crotonylation-related genes with OA.

Gene	Probe ID	topSNP	SMR association			OR (95%CI)	HEIDI test	
			*b*	se	*P*		*P*	nSNPs
*ACOX3*	ENSG00000087008	rs10002453	0.009	0.040	0.820	1.009 (0.934, 1.091)	0.135	20
*YEATS2*	ENSG00000163872	rs4490385	−0.050	0.058	0.383	0.951 (0.850, 1.065)	0.510	20
*GCDH*	ENSG00000105607	rs12611068	0.047	0.022	0.032	1.048 (1.004, 1.093)	0.860	20

b, beta; se, standard error; P, *p*-value; SMR, summary-data-based Mendelian randomization; OA, osteoarthritis; SNP, single nucleotide polymorphism; OR, odds ratio; HEIDI, heterogeneity in dependent instrument.

### 3.3 DEG analysis based on RNA-seq

**Figure 3:**
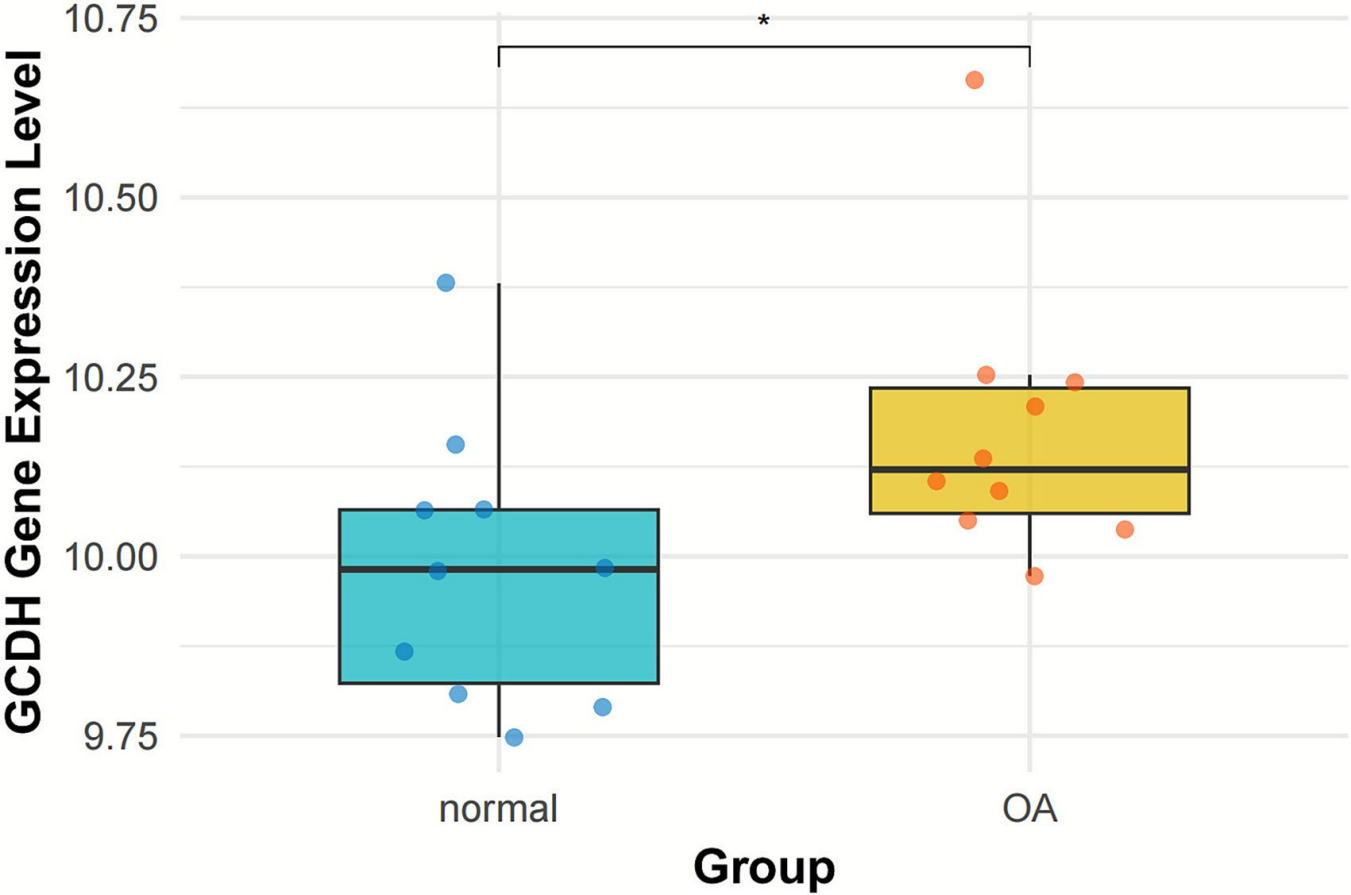
GCDH gene expression in normal and OA cartilage retrieved from GEO datasets. GCDH, Glutaryl-CoA dehydrogenase; OA, osteoarthritis; GEO, Gene Expression Omnibus. *p < 0.05.

RNA-seq data from both OA-affected and normal knee articular cartilage were extracted from the GSE117999 dataset. DEG analysis indicated that the expression level of the *GCDH* gene was notably elevated in OA patients relative to healthy controls (*p* < 0.05) (Figure 3).

### 3.4 Validation of the genetic expression of GCDH

**Figure 4:**
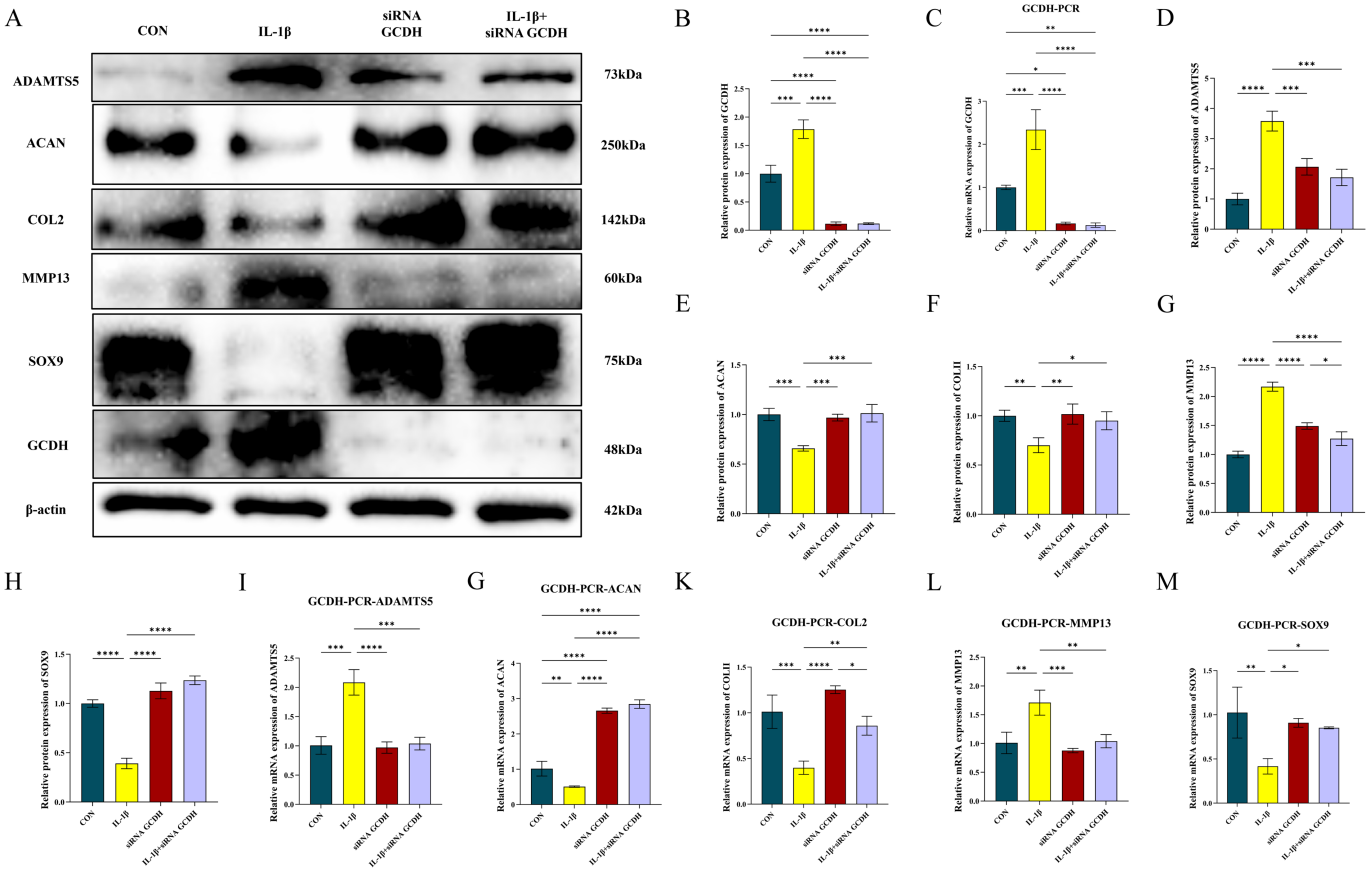
Evaluation of GCDH expression and regulation of key extracellular matrix markers via Western blot and qRT-PCR in OA chondrocytes. **(A–H)** Western blot analysis of GCDH, ADAMTS5, Aggrecan, COL2A1, MMP13, and SOX9 protein expression in normal and IL-1β-induced OA chondrocytes with or without GCDH knockdown. **(I–M)** qRT-PCR analysis of mRNA expression for GCDH, ADAMTS5, Aggrecan, COL2A1, MMP13, and SOX9 in normal and IL-1β-induced OA chondrocytes with or without GCDH knockdown. GCDH, Glutaryl-CoA Dehydrogenase; OA, osteoarthritis; qRT-PCR, quantitative real-time polymerase chain reaction. *p < 0.05, **p < 0.01, ***p < 0.001, ****p < 0.0001.

To validate the above findings, we examined the expression of *GCDH* in chondrocytes at both the protein and transcriptional levels. WB revealed that the protein expression level of *GCDH* was significantly upregulated in OA chondrocytes compared to normal controls, showing a 0.79-fold increase (*p* < 0.001) (Figures 4A,B). Consistent with this, qRT-PCR analysis demonstrated a marked 1.34-fold increase in GCDH mRNA expression levels (*p* < 0.001) (Figure 4C). Transfection with siRNA effectively suppressed *GCDH* gene expression in healthy and OA chondrocytes, and the knockdown efficiency was confirmed by both qRT-PCR and WB (Figures 4B,C).

### 3.5 GCDH regulates extracellular matrix metabolism in chondrocytes

To further investigate the role of *GCDH* in the regulation of ECM metabolism, we examined the expression changes of key markers associated with matrix synthesis and degradation. IL-1β stimulation significantly promoted the expression of catabolic markers ADAMTS5 and MMP13 at both protein and mRNA levels, while simultaneously suppressing the expression of anabolic markers Aggrecan, COL2A1, and the transcription factor SOX9 (Figures 4D–M). Notably, *GCDH* knockdown markedly reversed these IL-1β-induced aberrant expression patterns, restoring the levels of these markers to near-normal conditions.

### 3.6 Validation of the genetic function of GCDH

**Figure 5:**
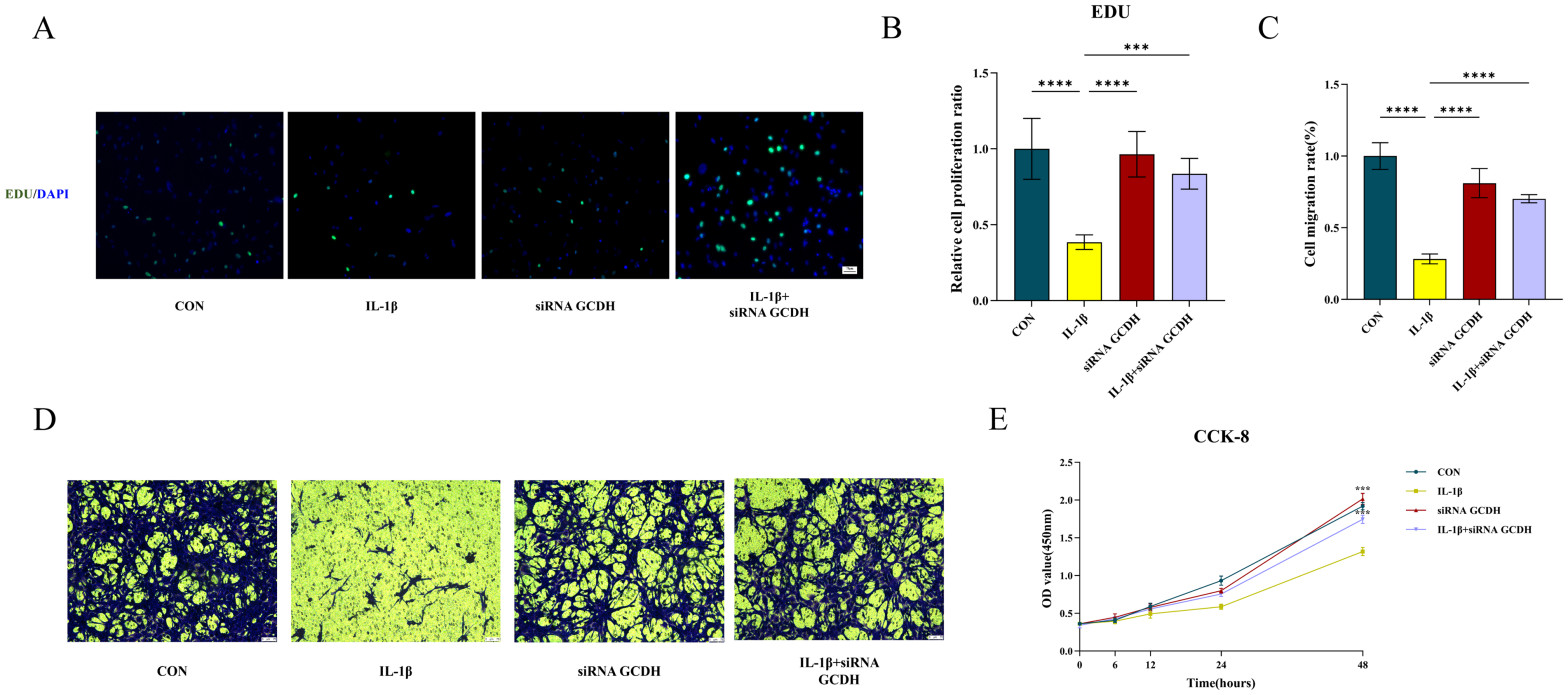
Functional experiments of GCDH gene based on chondrocytes. **(A,B,E)** Edu and CCK8 assays for the proliferation ability of chondrocytes in normal and IL-1β-induced OA chondrocytes with or without GCDH knockdown. **(C,D)** Transwell assay for migration behavior of chondrocytes in normal and IL-1β-induced OA chondrocytes with or without GCDH knockdown. GCDH, Glutaryl-CoA Dehydrogenase; OA, osteoarthritis; EdU, 5-Ethynyl-2′-deoxyuridine; CCK8, Cell Counting Kit-8. *p < 0.05, **p < 0.01, ***p < 0.001, ****p < 0.0001.

Subsequent functional experiments were conducted to assess chondrocyte proliferation and migration capacities. EdU assays revealed suppressed cellular viability in OA chondrocytes (*p* < 0.0001), while *GCDH* knockdown markedly increased EdU-positive cells in OA chondrocytes, indicating enhanced proliferative activity (*p* < 0.001) (Figures 5A,B). Consistent with these findings, CCK-8 assays demonstrated that IL-1β-induced chondrocytes exhibited significantly impaired proliferation compared to controls (*p* < 0.001). Notably, *GCDH* knockdown in OA chondrocytes restored their proliferative potential (*p* < 0.001) (Figure 5E). Furthermore, Transwell migration assays showed that *GCDH* knockdown effectively reversed the OA-associated decline in chondrocyte migratory capacity (*p* < 0.0001) (Figures 5C,D).

### 3.7 Downstream mechanisms

Previous studies have suggested that crotonylation modifications participate in various pathological processes by regulating metabolite production (29, 30). Notably, OA progression has been found to be closely associated with metabolic dysregulation (31). We therefore hypothesized that metabolites might serve as key downstream mediators through which *GCDH* promotes OA pathogenesis. Further mechanistic investigations revealed the specific metabolic pathway involved. Two-step MR analysis demonstrated that the crotonylation-related gene *GCDH* downregulates the expression of the plasma metabolite flavin adenine dinucleotide (FAD), thereby attenuating its protective effects against OA. In particular, FAD plays a significant role (10.7% mediation effect) in the causal pathway from *GCDH* to OA (Table 2).

**Table 2:** Mediating effect of flavin adenine dinucleotide on the risk of GCDH-osteoarthritis (OA).

Exposure	Mediator	Total effect	A	B	Indirect effect	Mediation effect
		β 3	β 1	β 2	β 1_2	(%)
GCDH	Flavin adenine dinucleotide (FAD) levels	0.048	−0.121	−0.043	0.005	10.7%

A, the effect of exposure (GCDH) on mediation (FAD); B, the effect of mediation (FAD) on outcome (OA) is independent of the effect of the exposure (GCDH); The formula “β1_2 = β 1 * β 2” was utilized to evaluate the indirect effect of mediating factors on OA, and “β1 * β2/β3” was utilized to calculate its mediating ratio.

## 4 Discussion

Once OA reaches end stage, it frequently becomes a predominant contributor to long-term disability within aging populations (32). Therefore, it is crucial to identify early interventions and treatments to delaying OA progression (33). This study integrates eQTL and GWAS data with MR analysis and *in vitro* experiments to systematically investigate the dynamic alterations and downstream regulatory mechanisms of *GCDH* genes during OA pathogenesis. Our findings will provide novel strategies for developing precision therapies targeting OA-associated crotonylation and facilitating early intervention.

In our research, *GCDH* was recognized as a potential risk element for OA pathogenesis through dual validation of OA-related crotonylation genes using the MR and SMR models. Meanwhile, significant upregulation of the *GCDH* gene in the OA group was observed in the DEGs analysis based on RNA-seq data, qRT-PCR, and WB results. Furthermore, *GCDH* was identified as a critical regulator of ECM metabolism in OA pathogenesis. Additionally, functional experiments indicated that, following *GCDH* knockdown, the proliferation and migration abilities of chondrocytes in the OA group were restored. Ultimately, the investigation of downstream mechanisms revealed that the plasma metabolite FAD mediated this process.

Kcr represents a novel PTM mechanism wherein crotonyl groups are covalently attached to lysine residues, thereby modulating protein functionality and cellular physiological processes (29, 34). Although no studies have directly elucidated the specific mechanisms of crotonylation-related genes in OA pathogenesis, accumulating evidence suggests their potential involvement in OA pathological progression through multiple pathways. Acyl-CoA synthetase short-chain family member 2 (*ACSS2*) has been shown to significantly elevate histone crotonylation levels, a modification that is closely linked to IL-1β-induced cellular fibrosis (35). Articular fibrosis is a significant characteristic of cartilage degeneration in OA (36). This observation indicates that crotonylation modifications may exacerbate the progression of OA by promoting fibrotic changes. Furthermore, Yu Zou et al. demonstrated that exogenous crotonyl coenzyme A can induce a significant pain response, which is associated with rapid upregulation of local pro-inflammatory cytokines TNF-*α* and IL-1β in the joints within 2 h post-injection (16). This mechanism suggests that up-regulation of crotonylation modification may be an important molecular basis for OA pain and dysfunction by promoting neuroinflammation and motor neurodegeneration.

Our study found that *GCDH* primarily promotes OA by altering the levels of the plasma metabolite FAD. Specifically, the expression level of *GCDH* is negatively correlated with plasma FAD, which is thought to delay the progression of OA. This finding suggests that the crotonylation-related gene *GCDH* may inhibit multiple protective pathways of FAD in the joints, thereby manifest as a risk factor for OA.

In recent years, nutritional and dietary interventions have been widely regarded as one of the most feasible strategies for the prevention and management of chronic diseases, owing to their universality and accessibility. Since B vitamins cannot be stored in large quantities in the body and require continuous daily dietary supplementation, the maintenance of their homeostasis is an indispensable aspect of nutritional supplementation (37). Among them, riboflavin (vitamin B_2_) plays a particularly critical metabolic role, as it is converted *in vivo* into two active coenzyme forms: flavin mononucleotide (FMN) and FAD. FAD serves as an essential cofactor for various flavoproteins and acts as a key regulator of mitochondrial energy metabolism—including the tricarboxylic acid cycle and the electron transport chain, as well as cellular redox homeostasis (38). Its function is realized through the cyclic interconversion of the FAD/FADH₂ redox couple, thereby driving multiple biological oxidation reactions, including mitochondrial respiration. Moreover, FAD acts directly as a cofactor in the transmission of genetic metabolic signals, playing crucial roles in processes such as nucleotide biosynthesis and tRNA methylation (39). Therefore, cellular FAD levels, which are determined by riboflavin intake, directly influence core physiological processes in chondrocytes, including energy metabolism, antioxidant defense, and inflammatory signal transduction.

The *GCDH* gene encodes glutaryl-CoA dehydrogenase, a key enzyme regulating crotonylation modification, which catalyzes the oxidation of glutaryl-CoA to crotonyl-CoA (34). This enzyme exists as a tetramer, with each subunit binding a molecule of FAD as a cofactor (40). Therefore, FAD depletion due to *GCDH* overexpression may drive the onset and progression of OA through multiple mechanisms, including mitochondrial dysfunction, exacerbated oxidative stress, and the promotion of chronic inflammation. A randomized controlled trial in OA mice demonstrated that supplementation with nutrients such as vitamin B_2_ significantly reduced OA incidence by 35%, suggesting that FAD deficiency may be a critical pathogenic factor in OA (41). Furthermore, FAD maintains mitochondrial membrane stability by regulating the Bcl-2/Bax ratio, thereby suppressing apoptosis dysregulation (42). Thus, when *GCDH* upregulation leads to excessive riboflavin consumption, it may promote mitochondrial dysfunction in chondrocytes, thereby accelerating OA progression. Moreover, as a crucial redox cofactor, FAD effectively maintains intracellular redox homeostasis by regulating the activity of antioxidants such as reduced glutathione (38). This mechanism significantly inhibits the overproduction of reactive oxygen species (ROS), thereby attenuating oxidative stress-induced chondrocytes impairment (43). Notably, in OA patients, abnormally elevated *GCDH* expression leads to a marked decrease in FAD levels, consequently impairing cellular defense against oxidative stress. Substantial evidence demonstrates that FAD suppresses neutrophil infiltration into joint tissues and attenuates the secretion of inflammatory factors TNF-*α* and IL-1β by modulating the NF-κB signaling pathway (44, 45). Given that persistent low-grade inflammatory response is widely recognized as a core pathological feature of OA, FAD deficiency leads to sustained activation of inflammatory responses in the chondrocyte microenvironment, ultimately exacerbating OA progression (46).

Our study possesses several notable strengths. Primarily, our work establishes crotonylation-associated genes as novel biomarkers for OA pathogenesis, offering potential therapeutic targets for early clinical intervention. Furthermore, the study integrates data analysis with *in vitro* cellular experiments, and both gene expression and functional assays further validate the reliability and accuracy of crotonylation-related genes in mediating OA pathogenesis. Several limitations of this study should be acknowledged. First, the primary reliance on GWAS data from European-ancestry populations necessitates further validation of our findings in other ethnic groups. Second, the relatively small sample size of the RNA-seq analysis requires confirmation in larger independent cohorts. Additionally, the lack of detailed clinical severity grading in public genomic databases highlights an important area for future research. In terms of experimental design, while the IL-1β-induced chondrocyte model effectively mimics key inflammatory aspects of osteoarthritis, it does not fully recapitulate the complexity of native joint tissue, including cell-matrix interactions and mechanical loading. Future studies will address these gaps by incorporating animal experiments. It should also be noted that the present study primarily focused on establishing the causal relationship between crotonylation-related genes and OA. A comprehensive proteomic analysis of protein crotonylation levels, along with in-depth validation of the underlying molecular mechanisms, would require substantial financial resources and advanced technical platforms, which were beyond the scope and capacity of the current investigation. Finally, identifying specific crotonylation targets in osteoarthritis and elucidating the precise molecular mechanisms by which *GCDH* regulates FAD biogenesis and signaling are crucial for understanding the downstream mediators of this pathway.

## 5 Conclusion

The crotonylation-related gene *GCDH* has been recognized as a potential contributor to OA pathogenesis, demonstrating not only upregulated expression during OA progression but also significantly correlated with the degradation of ECM of chondrocytes and the impairment of cell proliferation and migration capabilities. Notably, the plasma metabolite FAD has been shown to mediate the effects of *GCDH* on OA development. Our study elucidates the mechanistic link between crotonylation-associated genes and OA pathogenesis, providing novel insights for developing targeted therapeutic interventions against OA.

## Data Availability

The original contributions presented in the study are included in the article/Supplementary material, further inquiries can be directed to the corresponding author.

## Footnotes

1
https://www.ncbi.nlm.nih.gov/geo
