# Supplementary material for: Crotonylation-related gene GCDH promotes osteoarthritis pathogenesis through flavin adenine dinucleotide signaling: mechanism exploration and experimental validation
Source: Front Nutr. 2026 Jan 6;12:1646005. doi: 10.3389/fnut.2025.1646005 (PMC12815788; doi:10.3389/fnut.2025.1646005)
Supplement: Supplementary file 2 [file Supplementary_file_2.docx]

**Supplementary material 2 The primers of genes used in the article**

| Gene | Primer |
| --- | --- |
| *GCDH*-F | 5ʹ- CCT TGT CAT GCA CCC CAT CT-3ʹ |
| GCDH-R | 5ʹ- AGC CCA GAA GTT CAC CCT TG-3ʹ |
| ACAN-F | 5’-CGT GTA AAA AGG GCA CAG CC-3’ |
| ACAN-R | 5’-GGA AGC TCT TCT CAG TGG GC-3’ |
| COL2-F | 5’-GCC ATG ATT CGC CTC GG-3’ |
| COL2-R | 5’-CGG CTT CCA CAC ATC CTT ATC-3’ |
| ADAMTS5-F | 5’-GGC TCA CGA AAT CGG TAA CTG-3’ |
| ADAMTS5-R | 5’-GCA GGC AGA TTC TCC CCT TT-3’ |
| MMP13-F | 5’-GCA CTT CCC ACA GTG CCT AT-3’ |
| MMP13-R | 5’-AGT TCT TCC CTT GAT GGC CG-3’ |
| SOX9-F | 5’-GGA CCA CCC GGA TTA CAA GT-3’ |
| SOX9-R | 5’-AAG ATG GCG TTG GGG GAGAT-3’ |
| GADPH-F | 5’-GAC ATG CCG CCT GGA GAA AC-3’ |
| GADPH-R | 5’-AGC CCA GGA TGC CCT TTA GT-3’ |
