# Supplementary material for: Crotonylation-related gene GCDH promotes osteoarthritis pathogenesis through flavin adenine dinucleotide signaling: mechanism exploration and experimental validation
Source: Front Nutr. 2026 Jan 6;12:1646005. doi: 10.3389/fnut.2025.1646005 (PMC12815788; doi:10.3389/fnut.2025.1646005)

**Supplementary material 3 The sensitivity analysis of *GCDH, ACOX3,* and *YEATS2* gene expression and osteoarthritis**

1. The sensitivity analysis of *GCDH* gene expression and osteoarthritis


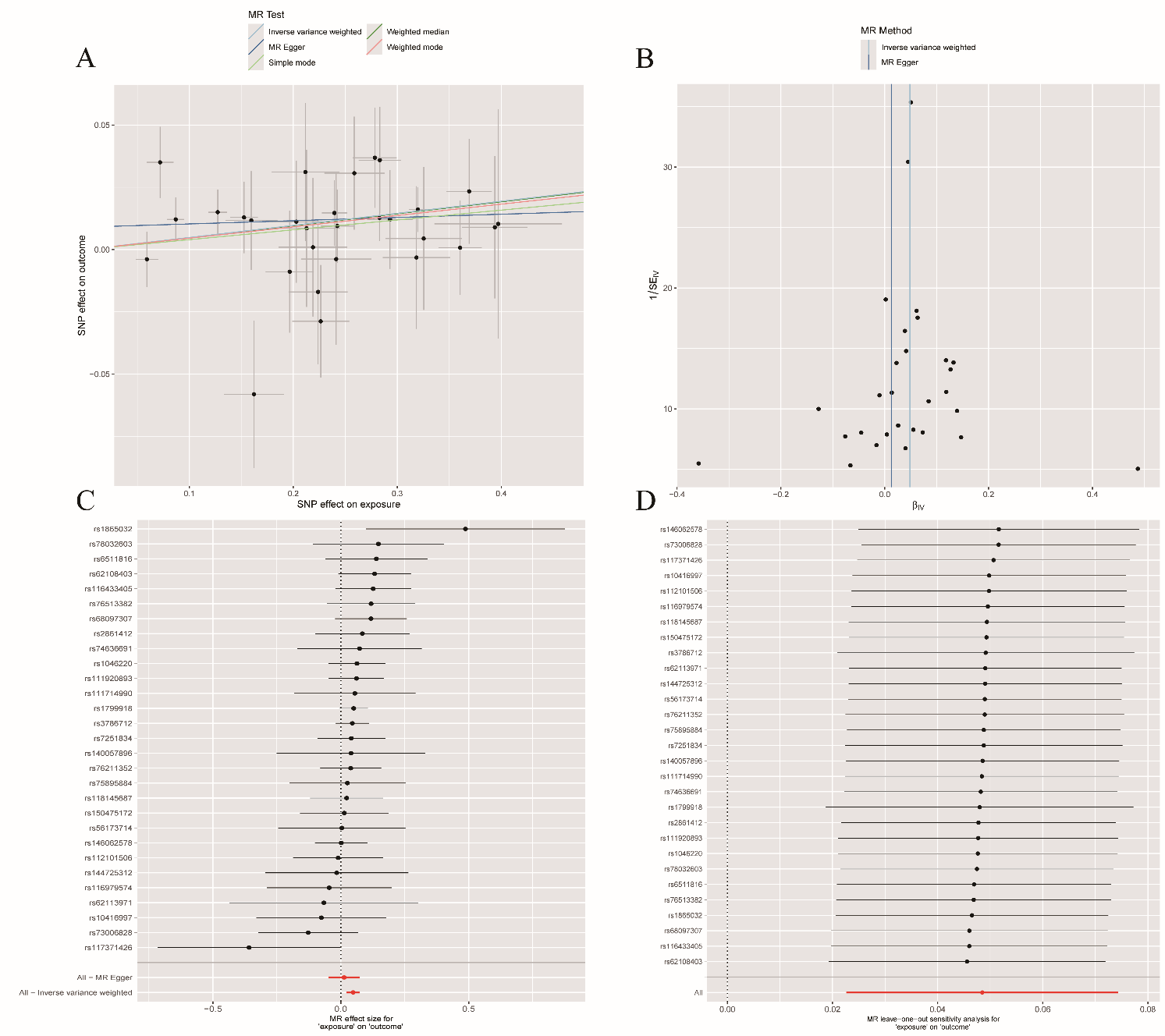


1. The sensitivity analysis of *ACOX3* gene expression and osteoarthritis


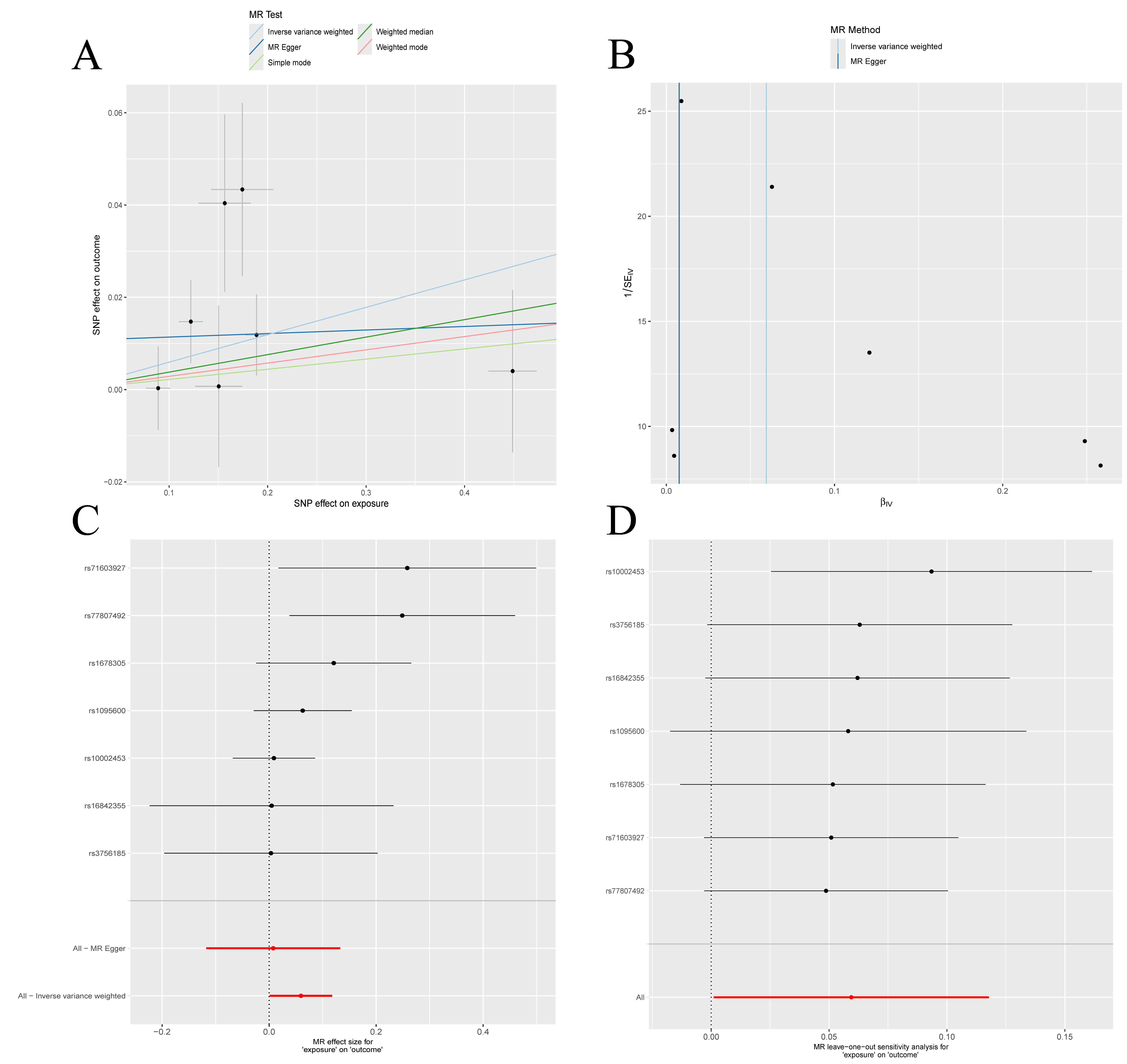


1. The sensitivity analysis of *YEATS2* gene expression and osteoarthritis


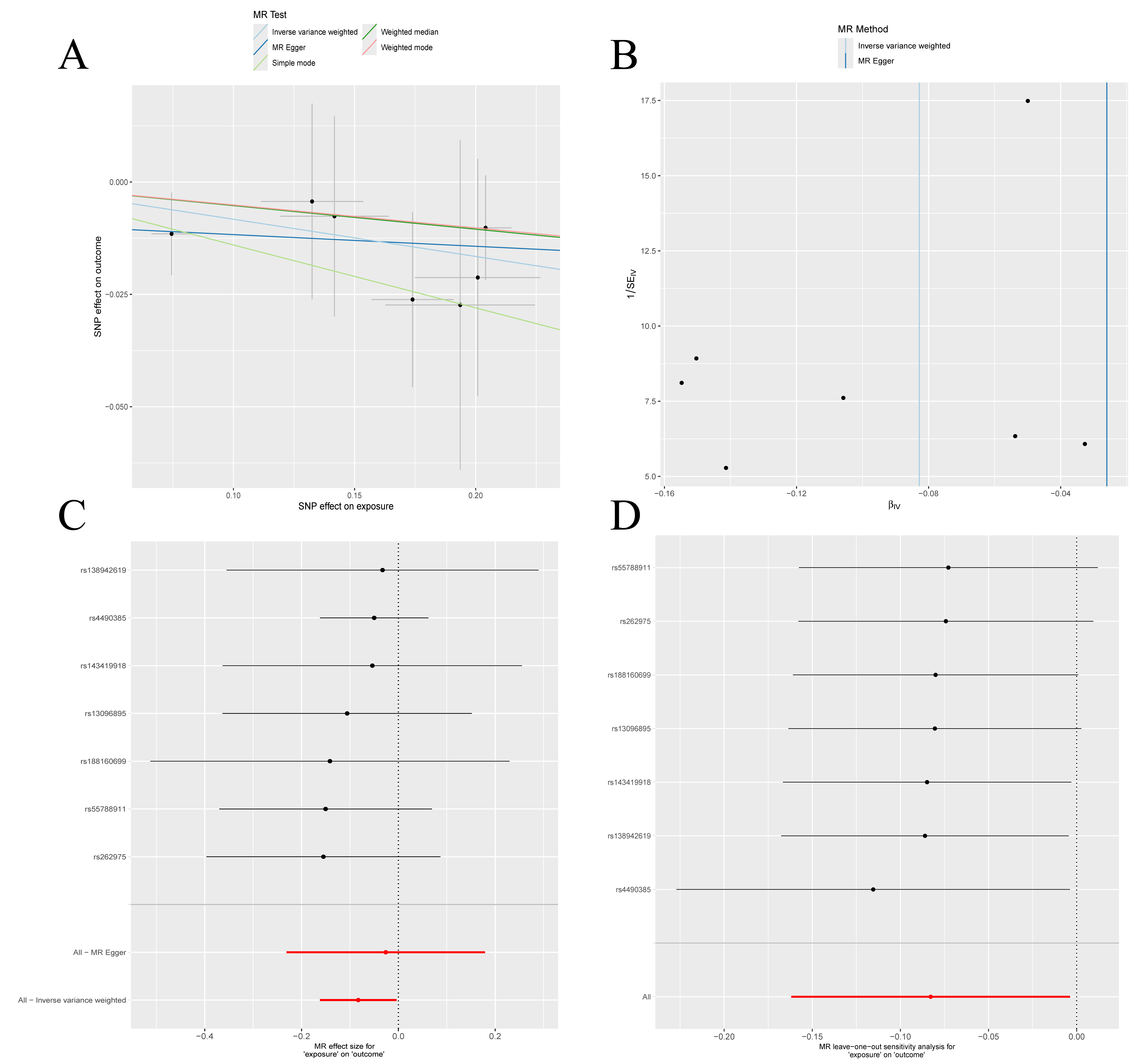

Supplement: Supplementary file 3 [file Supplementary_file_3.docx]
